# Calculation of Left Ventricular Relaxation Time Constant-Tau in Patients With Aortic Regurgitation by Continuous-Wave Doppler

**DOI:** 10.2174/1874192400802010028

**Published:** 2008-04-25

**Authors:** Xufang Bai

**Affiliations:** Henderson Research Center, McMaster University, Hamilton, Ontario, L8V 1C3, Canada

## Abstract

Left ventricular relaxation time constant, Tau, is the best index to evaluate left ventricular diastolic function. The measurement is only available traditionally in catheter lab. In Echo lab, several methods of non-invasive measurement of Tau have been tried since 1992, however almost all the methods are still utilizing the same formula to calculate Tau as in catheter lab, which makes them inconvenient, time-consuming and sometimes not very accurate. A simple method to calculate Tau in patients with mitral regurgitation has been developed just based on Weiss’ formula and simplified Bernoulli’s equation. Similarly, formulas are developed here by pure mathematical derivative to calculate Tau by continuous-wave Doppler in patients with aortic regurgitation.

## BACKGROUND

In some points of view, diastolic function of the left ventricle is more important for the abnormality usually appears ahead of the abnormal systolic function. In 1976, Weiss *et al. *[1] found that left ventricular pressure was able to be plotted and fit to an exponential function:

where P is the left ventricular pressure, e=2.71828…, is the natural logarithmic base, t is time from –dP/dt max, T is left ventricular relaxation time constant, also called Tau, and B is a constant. From Eq. (1), the derivative of the left ventricular pressure is expressed by:

Since P and -dP/dt are variables that can be obtained in catheter lab, the important parameter, Tau, becomes available through a simple calculation as shown above and its accuracy can be ensured through the accessibility of P and -dP/dt in the catheter lab. Obviously this method is inconvenient and invasive. Since 1992 many methods of noninvasive measurement of Tau by continuous-wave Doppler in patients with mitral regurgitation [2-5] or aortic regurgitation [6] have been reported. However, the derivation of the important parameters seems time-consuming and complicated *via *the proposed traditional method in Echo lab. I have introduced a simple method [7] to calculate Tau in patients in mitral regurgitation by pure mathematical derivative based on Weiss’ formula and simplified Bernoulli’s equation. Here a similar method is developed to calculate Tau by continuous-wave Doppler in patients with aortic regurgitation.

Before mathematical deduction, let’s refresh our knowledge about continuous-wave Doppler aortic regurgitation spectrum. Fig. (**1**) is a schematic description of aortic regurgitation spectrum.

From Fig. (**1**) we see the typical aortic regurgitation spectrum bordered by AVC-MVO-MVC-AVO, X axis is sweep time, the unit is ms. Y axis is aortic regurgitation spectrum velocity, the unit is m/s. Suppose there is no mitral opening, we can expect the ascending limb and the descending limb of the spectrum connected smoothly like the upper dash line. If we can add the mitral spectrum in the mean time, we will see the E spectrum and the A spectrum are within the MVO and the MVC. Time interval from AVC to MVO is isovolumic diastolic period. Time interval from MVC to AVO is isovolumic systolic period. For the ascending limb, the upper part could be bending more, making the (t3-t1)/(t2-t1)>>2. From MVO to MVC, the spectrum border is like a straight line, but actually it is not. According to simplified Bernoulli’s equation, the velocity is dictated by the pressure gradient between ADP and LVDP, ADP is aortic diastolic pressure, LVDP is left ventricular diastolic pressure:

For this LVDP is measured during mitral valves opening, we have LVDP<< ADP. Roughly, we have:

Whenever there is a severe aortic regurgitation, ADP decreases quickly, causing rapid decreasing of v. That’s why we classify the severity of aortic regurgitation with the help of pressure half-time and slope of MVO-MVC.

–dp/dt max happens shortly after AVC [1]. Or there is a short time delay between AVC and –dp/dt max. [8,9] In addition, after the return of pressure to the level of end-diastolic pressure, passive viscoelastic properties may be of importance and its effect on the evaluation of Tau should be modeled. [1] In another word, neither the beginning nor the end of this period fits the function P=E TOCMJ-2-28-img-01 very accurately, although the middle part of the isovolumic diastolic period does.

## DEDUCTION OF FORMULAS

**Fig. (1):**
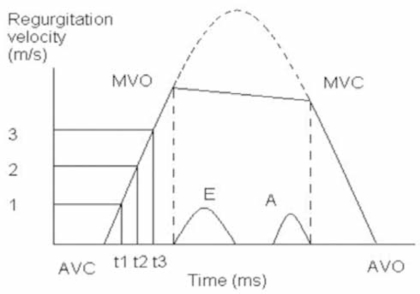
Schematic description of aortic regurgitation spectrum. AVC is aortic valves closure, MVO is mitral valves opening, MVC is mitral valves closure and AVO is aortic valves opening.

In the Echo examination for patients with aortic regurgitation, the left ventricular pressure, P, can be expressed as:

where ΔP is the pressure gradient between aorta and left ventricle. Substituting Eq. (1) and the simplified Bernoulli’s equation: ΔP=4v²into Eq. (4) leads to the following equation:

A natural logarithmic transformation on both sides of the above equation results in the expression:

Three points, (t1, 1m/s), (t2, 2m/s) and (t3, 3m/s), are chosen on the ascending limb of the aortic regurgitation continuous-wave Doppler velocity curve (Fig. 1), and substituted into Eq. (6) respectively, which can come across the following three equations:

From the difference comparison of Eqs. (7a) and (7b), we can find:

Similarly,

From the above formulas (8a) and (8b), both Tau and ADP can be calculated after we measure two time intervals: (t3-t1) and (t2-t1). Fig. (1) shows how measurement is done.

## ADVANTAGES OF THE METHOD

The deduction based only on Weiss’ formula, simplified Bernoulli’s equation, the pure mathematical derivative makes the method almost universal. For calculation of Tau and ADP based on of Eq. (8a) and (8b), a simple computer program can be developed to make the calculation easier. The method is applicable to all patients with acceptable quality of aortic regurgitation spectra only if the application of the Weiss’ formula and simplified Bernoulli’s equation are acceptable.

## DERIVATION OF ADP

**Fig. (2):**
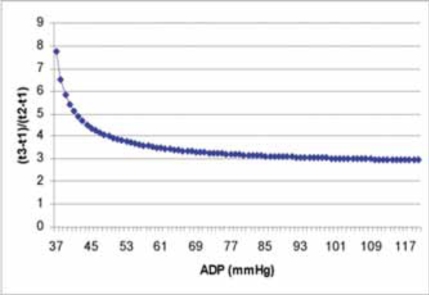
The curve derived from Eq. (9) shows the relation of (t3-t1)/(t2-t1) and ADP.

Calculation of the ratio of Eqs. (8a) and (8b) leads to the following equation:

from this equation we can draw the conclusion that ADP is determined by the ratio of (t3-t1)/(t2-t1) on the ascending limb of aortic regurgitation continuous wave Doppler spectrum. (Fig. **2**) Interestingly, when (t1-t3)/(t1-t2)=3 is considered, from Eq. (9), we can find the corresponding result, ADP is around 108 mmHg. When (t3-t1)/(t2-t1)=4, ADP is around 49 mmHg. Which means when ADP is lower, the ascending limb of the aortic regurgitation curve bends more. Further study about this relationship between (t3-t1)/(t2-t1) and ADP must be very exciting and fruitful.

## CAN WE CALCULATE TAU *VIA *(v3-v1) IN AORTIC REGURGITATION PATIENTS LIKE WE HAVE THE FORMULA TAU=1.2(t1-t3) IN MITRAL REGURGATION PATIENTS?

From Eq. (8b) Tau= (t3-t1)/ln((ADP-4)/(ADP-36)), we have:

To better understand the relation of ADP and Tau/(t3-t1), Fig. (**3**) is plotted based on Eq. (10).

From Fig. (**3**), we can see the ADP covers a wide range and the corresponding Tau/(t3-t1) changed a lot accordingly. It is difficult to develop a formula to calculate Tau *via *(t3-t1) in aortic regurgitation patients like we have the formula Tau=1.2(t1-t3) in mitral regurgitation patients. [7]

## LIMITATIONS

Only data for patients with aortic regurgitation and fairly good quality of Doppler spectra can be used to calculate Tau. Therefore, for most the patients in clinic, the noninvasive measurement of Tau and ADP is still inapplicable.

## FUTURE ENDEAVOR

When we tried to calculate ADP from (t3-t1) and (t2-t1), we presumed the ADPs were the same at the 3 time points: 3m/s, 2m/s and 1m/s. In fact, some difference does exist among the 3 time points. If more precise measurement methods are available, it is possible to choose the 3 points more closely, such as 2.1m/s, 2m/s and 1.9m/s, which is helpful to decrease the systematic error caused by ADP variation.

In this digital era, the spectra border has already been digitized, we can expect integration of these formulas (8a) and (8b) into an Echo machine will enable a simple and effective derivation of Tau and ADP immediately after we get an aortic regurgitation continuous-wave Doppler spectrum.

Further investigations with large sample number from primary patient data will be helpful to fully prove the method.

## Figures and Tables

**Fig. (3):**
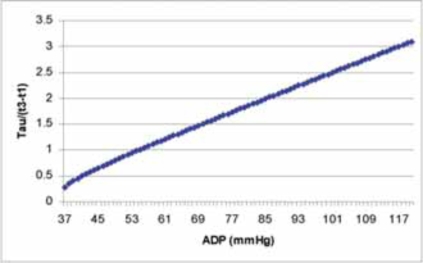
The curve derived from Eq. (10) shows the relation of Tau/(t3-t1) and ADP.
